# Attitudes toward pharmacological cognitive enhancement—a review

**DOI:** 10.3389/fnsys.2014.00053

**Published:** 2014-04-17

**Authors:** Kimberly J. Schelle, Nadira Faulmüller, Lucius Caviola, Miles Hewstone

**Affiliations:** ^1^Department of Industrial Design, Eindhoven University of TechnologyEindhoven, Netherlands; ^2^Department of Experimental Psychology, University of OxfordOxford, UK; ^3^Department of Values, Technology and Innovation, Delft University of TechnologyDelft, Netherlands

**Keywords:** cognitive enhancement, neuroenhancement, brain function augmentation, medical safety, coercion, fairness, authenticity, smart drugs

## Abstract

A primary means for the augmentation of cognitive brain functions is “pharmacological cognitive enhancement” (PCE). The term usually refers to the off-label use of medical substances to improve mental performance in healthy individuals. With the final aim to advance the normative debate taking place on that topic, several empirical studies have been conducted to assess the attitudes toward PCE in the public, i.e., in groups outside of the academic debate. In this review, we provide an overview of the 40 empirical studies published so far, reporting both their methodology and results. Overall, we find that several concerns about the use of PCE are prevalent in the public. These concerns largely match those discussed in the normative academic debate. We present our findings structured around the three most common concerns: medical safety, coercion, and fairness. Fairness is divided into three subthemes: equality of opportunity, honesty, and authenticity. Attitudes regarding some concerns are coherent across studies (e.g., coercion), whereas for others we find mixed results (e.g., authenticity). Moreover, we find differences in how specific groups—such as users, nonusers, students, parents, and health care providers—perceive PCE: a coherent finding is that nonusers display more concerns regarding medical safety and fairness than users. We discuss potential psychological explanations for these differences.

## Introduction

Brain enhancement is a topic of huge interest in the media and the academic literature. An often discussed form of brain enhancement is *pharmacological cognitive enhancement* (PCE) which usually refers to the use of certain prescription substances. Schermer et al. (2009) define these sorts of enhancements as “pharmacological interventions that are intended to improve certain mental functions and that go beyond currently accepted medical indications” (p. 77). PCE is not only a topic that is debated in academia—mainly in neuroscience, law, and ethics—but also a reality: several surveys show that students, but also other groups such as academics and surgeons, use substances to enhance their cognitive performance (e.g., Maher, 2008; Smith and Farah, 2011; Franke et al., 2013). Examples of seemingly common pharmacological enhancers are methylphenidate (e.g., Ritalin®), mixed amphetamine salts (e.g., Aderall®), and modafinil (e.g., Provigil®). Originally, the first two were developed as treatment for Attention Deficit Hyperactivity Disorder and the latter as treatment for Narcolepsy, but now they are being used to enhance performance in healthy individuals.

Although there are considerable intra- and interpersonal differences in the effects of these substances (e.g., Husain and Mehta, 2011; Van Der Schaaf et al., 2013), average effects have been reported. In their overview of findings and meta-analysis of the effects of methylphenidate and modafinil in healthy individuals, Repantis et al. (2010) assessed mood, motivation and four categories of cognitive processes: wakefulness, attention and vigilance, memory and learning, and executive functions and information processing. The available data about methylphenidate did not provide enough information to draw a firm conclusion about the effects of methylphenidate on enhancing or maintaining performance, although evidence for a positive effect on memory was found. An enhancing effect on attention was not verified, conflicting with the goal of increasing concentration and alertness that users of enhancement substances often have (Teter et al., 2005). The aggregated findings of potential enhancing effects of modafinil indicate that it improves attention for well-rested individuals. Furthermore, it helps in maintaining a higher degree of wakefulness, memory and executive functions over a period of sleep deprivation (Repantis et al., 2010). The authors suggest that these effects led to a growing popularity of modafinil, and strongly recommend a public debate on the ethics of the use of PCE which takes this into account. However, other reviews did not find any effect or even suggested that the non-cognitive effects of the substances, e.g., on confidence and motivation, might be responsible for enhanced performance (Farah et al., 2014).

In the normative debate about whether or not PCE is to be endorsed, certain concerns about its use are often raised and have been discussed by several authors (e.g., Farah et al., 2004; Bostrom and Sandberg, 2009; Schermer et al., 2009; Hyman, 2011). In this review, we focus on three concerns that are most often emphasized by the public, but are also discussed in the normative debate. First, the *medical safety* of the aforementioned substances has been discussed, in particular in terms of short- or long-term side effects. A second topic of discussion is *coercion*, relating to concerns about the explicit and implicit pressures that can arise from the availability of PCE, forcing people to use these substances to be able to compete, for example at the workplace or in school. The third concern relates to the *fairness* of the use of PCE. This concern includes several subtopics such as a possible unequal distribution of access to such enhancers, their use in competitive environments being seen as cheating, and also to what degree performance brought about under PCE can be seen as authentic.

There are good reasons to examine the *attitudes of the public* toward PCE in addition to this normative debate. In their plea for the inclusion of public views on developments in biomedicine and technology, Schicktanz et al. (2012) underline a view already suggested a few years ago by Sarewitz (2010) in a column in *Nature.* They propose three general arguments for the inclusion of the public in ethical reasoning. First, views and attitudes of the public can point to remote or emerging moral problems. Second, empirical research can be used to examine premises about human behavior and social consequences of actions that underlie several applied ethical arguments. Third, research on public opinions can increase the context-sensitivity of ethical reasoning by pointing out consequences of concrete decisions in social policy. They argue that any bioethical discussion that avoids a confrontation with public opinions “not only runs the risk of missing important aspects, ideas, and arguments. It also arouses strong suspicion of being indeed one-sided, biased or ideological—thus illegitimate.” (Schicktanz et al., 2012, p. 136). Apart from the aforementioned general arguments to include the public's attitude in certain bioethical discussions, specific arguments for the case of PCE are also made. Faulmüller et al. (2013) describe how PCE, as long as it is perceived negatively by the public, might give rise to indirect psychological costs for users: they might be treated in ways that damage their psychological well-being, for example by a misattribution of their success to the enhancer rather than themselves, or by dehumanization or ostracism. More insight into the public's view can lead to more insight into these potential indirect psychological costs. In addition, Banjo et al. (2010) argue that physicians are and will be “gatekeepers” in dispensing at least a portion of PCE substances. Although there is discussion on if and how their views might affect social policy or legal regulation on PCE (Delaney and Martin, 2011), PCE is part of their clinical reality. Therefore their views can provide specific insights that are of importance to the general debate (Ott et al., 2012).

The current article is based on an extensive literature search. We used two databases, Web of Science and Scopus, and included all articles published in English between 1990 and 2014. The search terms we used were [(“cognitive enhancer” OR “cognitive enhancement” OR “pharmacological enhancement” OR “prescription drug” OR “performance enhancing drug” OR neuroenhancement OR “human enhancement”) AND (view OR perspective OR opinion OR attitude OR judgment OR motive OR justification)] as part of the title, abstract or keyword of the text. This search resulted in 447 articles in Web of Science and 4162 articles in Scopus. Based on a careful check of titles and abstracts, we selected all articles that reported empirical studies on the opinions of groups outside the normative academic debate (such as students or physicians) on the use of PCE. We also cross-checked the reference lists of the studies found this way to identify additional relevant literature matching our inclusion criteria. Moreover, we checked Google Scholar and asked colleagues publishing in this area to direct us to relevant publications. Overall, this search resulted in 40 publications reporting qualitative or quantitative examinations of attitudes, opinions, and views of the general public and more often specific sub-populations toward PCE, which we included in this review. These 40 publications are marked with an asterisk in the reference list.

Our overarching finding is that in the public several concerns about the use of PCE substances are prevalent: they agree on several concerns when being asked specifically and raise concerns themselves when being asked for their opinion. These concerns widely match the normative debate. Hence, this review is structured around the common concerns reflected in the academic and public debate: (1) medical safety, (2) coercion, and (3) fairness. Each section in this review will be introduced with a short description of the academic debate on the respective concern. Then, the relevant empirical studies are reported. We also try to pinpoint some shortcomings in the current literature and suggest potential paths for future research. Table 1 provides an overview of key methodological aspects of all studies reviewed, containing information about the concerns investigated, the research method by which the data were obtained, the country where the study took place, and the sample (occupation of participants, sample size, sampling method, and response rate).

**Table 1:** **Overview of the empirical studies discussed in this review**.

**Authors**	**Country**	**Occupation of participants**	**Sampling method**	**Research method**	**Response rate**	**Sample size**	**Concerns**
Aikins, 2011	USA	University students	Purposive sampling	Semi-structured interview	n/a	12	Safety, fairness
Asscher and Schermer, 2013	The Netherlands	General public	Purposive sampling	Focus groups	n/a	37	Safety
Ball and Wolbring, 2014	Canada	Parents	Purposive sampling	Semi-structured interview	n/a	12	Safety
Banjo et al., 2010	USA and Canada	Physicians	Convenience sampling	Web-based survey	n/a	212	Safety, coercion, Fairness
Bell et al., 2013	Australia	University students	Convenience sampling	Interview	n/a	19	Safety, coercion
Bergström and Lynöe, 2008	Sweden	General public	Random sampling	Paper and pencil questionnaire	52%	517	Safety
		Physicians			39%	108	
Bossaer et al., 2013	USA	University students	All students at one university invited	Web-based survey	59.9%	372	Safety, fairness
Desantis and Hane, 2010	USA	University students	Convenience sampling	Interview	n/a	175	Safety
Dodge et al., 2012	USA	University students	All students at one university invited	Web-based survey	37%	±1200	Fairness
Dubljević et al., 2013^*^	Germany	University students	Three stage cluster sampling (universities, disciplines, students)	Web-based survey	First wave 53.5%	5882	Fairness
					Second wave 69.1%	3486	
Eickenhorst et al., 2012	Germany	University students	Convenience sampling	Web-based survey	n/a	1218	Safety
		University graduates				106	
European Citizens Panel, 2006	Belgium, Denmark, France, Germany, Greece, Hungary, Italy, the Netherlands, UK	General public	Stratified random sampling (age, profession, gender)	Citizen's deliberation	n/a	126	Coercion
Fitz et al., 2013	USA and Canada	General public	Convenience sampling, Amazon's Mechanical Turk recruitment	Web-based survey	n/a	4011	Safety, coercion, fairness
Forlini and Racine, 2009	Canada	University students	Purposive sampling	Focus groups	n/a	29	Coercion
		Parents				21	
		Health care providers				15	
Forlini and Racine, 2012a	Canada	University students	Purposive sampling	Focus groups	n/a	29	Safety, coercion, fairness
		Parents				21	
		Health care providers				15	
Forlini and Racine, 2012b	Canada	University students	Purposive sampling	Focus groups	n/a	29	Safety, fairness
		Parents				21	
		Health care providers				15	
Franke et al., 2012a^*^	Germany	High school students	All students at 12 public grammar and vocational schools, and students of three departments of one university invited	Paper and pencil questionnaire	83%	1035	Safety, coercion, fairness
		University students				512	
Franke et al., 2012b	Germany	University students	Convenience sampling	Interview	n/a	22	Safety, coercion, fairness
Franke et al., 2014	Germany	Physicians	All primary care physicians in one state invited	Paper and pencil questionnaire	30.2%	832	Safety
Hotze et al., 2011	USA	Physicians	Random sampling	Paper and pencil questionnaire	46.6%	633	Fairness
Judson and Langdon, 2009	USA	University students	All students at two colleges invited	Paper and pencil questionnaire	10%	333	Safety
Kudlow et al., 2013	Canada	University students	All medical students at one medical school invited	Web-based survey	50%	326	Safety
Maier et al., 2013	Switzerland	University students	All students at three educational institutions invited	Web-based survey	22.3%	6275	Coercion
Maslen et al., in press	Germany	University students	Convenience sampling	Paper and pencil questionnaire	n/a	80	Coercion
Mazanov et al., 2013	Australian	University students	Convenience sampling	Web-based survey	n/a	1729	General, fairness
Ott and Biller-Andorno, 2013	Switzerland	University students	Convenience sampling	Web-based survey and separate paper and pencil questionnaire	n/a	1765	Safety, fairness
Ott et al., 2012	Switzerland	Physicians	Stratified random sampling (profession, gender, years of training, language)	Paper and pencil questionnaire	23.9%	379	Safety
Partridge et al., 2012	Australia	General public	Random sampling	Telephone interview	31.9%	1265	General
Partridge et al., 2013	Australia	University students	Convenience sampling	Interview	n/a	19	Safety
Riis et al., 2008	USA	University students	*No information provided*	Web-based survey	n/a	357	Fairness
Sabini and Monterosso, 2005	USA	University students	Convenience sampling	Paper and pencil questionnaire	n/a	185	Fairness
Santoni de Sio et al., in press	United Kingdom	University students	Convenience sampling	Paper and pencil questionnaire	n/a	102	Safety, fairness
Sattler et al., 2013a^*^	Germany	University students	Three stage cluster sampling (universities, disciplines, students)	Web-based survey	87.1%	1852	Safety, fairness
(Sattler et al., 2013b)^*^	Germany	University teachers	Three stage cluster sampling (universities, disciplines, students/teachers)	Web-based survey	33.5%	1402	Safety
		University students			69.1%	3486	
Sattler et al., 2014^*^	Germany	University students	Three stage cluster sampling (universities, disciplines, students); only second time wave	Web-based survey	69.1%	3486	Safety, coercion, fairness
Sattler and Wiegel, 2013^*^	Germany	University students	Three stage cluster sampling (universities, disciplines, students); only second time wave	Web-based survey	First wave 53.5%	5882	Safety
					Second wave 69.1%	3486	
Scheske and Schnall, 2012	UK	University students	Convenience sampling, two studies - two samples	Paper and pencil questionnaire	n/a	50	Safety, fairness
						306	
Schildmann et al., 2013	Germany	University students	*No information provided*	Survey	n/a	1026	Coercion, fairness
Schuijff and Brom, 2013	The Netherlands	All	Purposive sampling	Focus groups	n/a	38	Safety, coercion, fairness
Sweeney, 2010	USA	University students	Convenience sampling	Paper and pencil questionnaire	n/a	100	Safety, fairness

Convenience sampling and purposive sampling require no random selection of participants, whereas random sampling, stratified random sampling and cluster sampling do. Purposive sampling requires obtaining a sample of people who meet a predetermined criterion, whereas convenience sampling does not. For stratified random sampling, a population is divided in strata (subgroups) from which participants are randomly selected to make sure all strata are represented in the sample in proportion to their prevalence in the population. Cluster sampling requires a list of clusters, e.g., disciplines in a university, from which a few clusters are randomly chosen. Instead of randomly selecting participants from a list of potential participants, e.g., all students of the university, every member of the selected cluster is invited to participate (Cozby, 2009).

* The authors explicitly state that N is not equal for each analysis due to missing data or specific criteria employed.

## Medical safety

The normative debate about the safety of PCE usually focuses on potential trade-offs between benefits and risks. As with all medical procedures, there might be side effects and yet-unknown long-term health risks with substances such as methylphenidate or modafinil (King et al., 2006). Schermer et al. (2009) point out that the harm-benefit ratio of PCE deserves special consideration, as the life-improving benefits might not outweigh the potential risks for consumers who use them for enhancement purposes, instead of the therapeutic purposes for which the substances were originally developed. However, Dubljevic (2013) emphasizes the importance of analyzing different substances in a case-by-case approach, as some entail greater health and addiction risks than others (Kociancic et al., 2004). Several studies have examined people's health concerns about such cognitive enhancers. Interestingly both users and nonusers systematically overestimate the cognitive-enhancing effects of PCE (Finger et al., 2013; Ilieva et al., 2013), but a sharp discrepancy between their risk estimations is revealed. While nonusers generally have strong concerns regarding the safety of PCE, users show less concern.

*Nonusers* tend to believe that such substances are addictive, might induce sleep disorders and may even lead to mental health problems, which was shown in an interview study with 19 Australian students (Partridge et al., 2013). In two surveys conducted on a UK university campus with a total of 357 students, Scheske and Schnall (2012) observed that moral reservations against PCE stem mostly from these safety concerns: the riskier the substances, the more student respondents morally object to their non-medical use. Similarly, in a survey involving 102 UK Science students, Santoni de Sio et al. (in press) found that the concern against enhancement use that was raised most often by respondents related to potential unintended side-effects.

A plausible consequence is that students are less willing to engage in PCE the higher the severity and risk of the resulting health issues are perceived to be. This was shown in two German factorial design online surveys based on a large pool of vignettes with a sample of 1852 students in the first study (Sattler et al., 2013a) and 3486 students and 1402 university teachers in the second study (Sattler et al., 2013b; these are results of the second wave of a biannual project; also see Sattler and Wiegel, 2013 and Dubljević et al., 2013 for findings on the first and second wave, and (Sattler et al., 2014) for other results on the second wave). Moreover, Franke et al. (2012a) showed in an extensive paper-and-pencil questionnaire study with a sample of 1547 German students that the majority would consider taking PCE substances only if their safety could be assured.

The relation between willingness to use PCE and the perceived risk of PCE might also be an explanation for the results from a focus group study on several human enhancement technologies conducted with 38 Dutch participants divided into five groups (Schuijff and Brom, 2013). (A focus group study is a qualitative research technique in which a group of participants discuss their opinions on a given topic.) After more information on the effects and risks of using methylphenidate as an enhancer was provided, fewer participants stated they would consider using the substance for enhancement purposes than before receiving the information. Participants in this study were not familiar with the concept of human enhancement, in contrast to participants in most of the other studies, and therefore might have underestimated the risks accompanying the use of PCE. In another Dutch focus group study with 37 participants divided into five groups, several examples of the use of medical means to fulfill non-medical wishes were investigated. An example related to PCE, taking β-blockers during a driving test, raised several concerns about medical risks (Asscher and Schermer, 2013). In a semi-structured interview study with twelve Canadian parents of either cognitively disabled or non-disabled children, Ball and Wolbring (2014) observed that all parents unanimously agreed that medical safety has to be insured in order to administer PCE to their children.

Sattler and Wiegel (2013) provided the first evidence on the influence of the perceived severity of side effects and risk attitudes on actual PCE use. In a large- scale online survey with 5882, in a first wave, and 3486 respondents, in a second wave, a lower proneness to risk and an expectation of more severe drug-related side effects were associated with more PCE use at the first time point and increased use of PCE over a 6-months period. However, other results in the second wave of the same project did not show the relation between the expected severity of side effects or risk attitudes and willingness to use PCE (Sattler et al., 2014). The authors did, though, find in the second wave that a higher expected likelihood of side effects decreased the willingness to use PCE.

Other findings suggest that natural remedies are perceived as less harmful than PCE substances, as shown in a questionnaire study involving the Swedish general public and physicians with a sample size of 625 in total (Bergström and Lynöe, 2008). Furthermore, people morally object more to the application of PCE substances if they are artificial rather than natural, and if they are taken in the form of injections rather than pills (Scheske and Schnall, 2012). Focus group participants in the Netherlands referred to natural remedies, placebo, and psychological treatment as alternatives to a specific example of PCE (taking β-blockers during a driving test; Asscher and Schermer, 2013). However, these results may depend on the familiarity of the sample with medical substances. An online survey among 326 Canadian medical students showed that there were no significant differences between attitudes toward pharmacological or natural supplements for cognitive enhancement (Kudlow et al., 2013).

Furthermore, 212 US American and Canadian physicians, reported in an online survey, being less comfortable prescribing PCE substances for non-medical use to young adults, compared to older patients (Banjo et al., 2010). Franke et al. (2014) confirmed these findings in a paper and pencil questionnaire study among 832 German physicians. In both studies, physicians worried about misuse and deemed PCE for young people to be unnecessary. Yet, this applied less to the treatment (i.e., medical use) of older patients. Consistent with these findings, an experimental vignette study conducted online with 4011 respondents found that the US American and Canadian public is more tolerant of side effects when they can be seen as the result of necessary therapy instead of enhancement (Fitz et al., 2013). This touches upon a big debate in medical ethics about the distinction between treatment and enhancement, since this is not a distinction based on biological facts, but, rather, reflecting subjective valuation (Parens, 1998; Daniels, 2000; Hyman, 2011). This is also reflected in a survey by Ott et al. (2012), who concluded that subjective suffering is taken into account as a criterion for disease. From the respondents, 379 Swiss general practitioners and psychiatrists, 88% reported being influenced by the degree of subjective suffering in prescribing an enhancer in four different scenarios. When asked if they would prescribe a PCE substance if a student requested a prescription to stay awake to study more, only 15% confirmed without any doubt, although 54% would prescribe if there were no therapeutic alternative. Around half of the respondents reported being confronted in their practice with such requests for prescriptions.

In contrast to these consistent findings on nonusers, *users* of PCE differ in their estimation of the safety of PCE substances. In an online survey with 1324 German students, users tended to rate the health consequences of PCE as less dangerous than nonusers did (Eickenhorst et al., 2012). This could be explained by users' higher ratings of willingness to take risks, found in a large-scale online survey involving 1765 Swiss students (Ott and Biller-Andorno, 2013). Moreover, fewer individuals (63.9%) from this latter survey's “user group” of 108 students reported worrying about side effects than individuals from the “nonuser group” of 1689 students (81.9%). Similarly, in a semi-structured interview study with 12 US American students who were illicitly or licitly using PCE, most participants believed that the benefits of PCE outweighed the potential negative side effects (Aikins, 2011). Another study with 333 US American students revealed that students who were illicitly using PCE had even more positive attitudes to the use of PCE with regard to medical safety issues compared to licit users (Judson and Langdon, 2009). In another interview study with 22 German students who used both caffeine and PCE, participants saw differences between the two forms of enhancement: in particular they estimated both the desired effects and the negative side effects as more pronounced in the case of PCE (Franke et al., 2012b). Also, Sweeney (2010) presents in her thesis results of a campus survey with a sample of 100 US American students, which demonstrate that students who are illicitly using cognitive enhancers seem to be more likely than nonusers to believe that the substances are harmless.

The aforementioned findings concerning differences between users and nonusers in their estimation of risks and harmfulness might also explain the large difference between the attitudes of users and nonusers found in other studies. A study on attitudes toward the acceptableness of PCE amongst 1265 members of the general public in Australia found that respondents who were familiar with PCE—either by using it themselves or by knowing somebody who used PCE—were twice as likely to find PCE acceptable than respondents who were not familiar with it (Partridge et al., 2012). Also users of PCE among a group of Canadian medical students tended to have more favorable attitudes toward PCE than nonusers (Kudlow et al., 2013). Lastly, a survey of 1729 students from Australian universities revealed differences in attitudes between users and nonusers of PCE, with users more often finding the use of study drugs “moral” than nonusers (Mazanov et al., 2013). The latter three studies did not specifically present the reason why respondents had a certain attitude toward PCE, and thus their opinions might not be related to medical safety. Still, these results are in line with a common finding in drug epidemiology, revealing that positive attitudes toward a drug, in particular low perceived risk, correlate with its use (e.g., Bachman et al., 1998; Bavarian et al., 2013; Cabriales et al., 2013).

In addition, users justify their practice and underestimate potential health risks for themselves and for others as shown in an interview-based survey with 175 US American students (Desantis and Hane, 2010). Conversely, Franke et al. (2012a) found that users and nonusers estimate addictive risks similarly, perhaps because there is a greater likelihood of substance dependency for users (Kroutil et al., 2006). However, Franke et al. (2012a) did not specifically test for PCE dependency in their study. In the above-mentioned study by Desantis and Hane (2010), it was revealed that users argue for the substances' safety and downplay potential health risks by contrasting PCE to street-drugs (e.g., cocaine, heroin, etc.) and by pointing toward their acceptance within the medical establishment. This relates to the observation that students who believe that they know enough in order to safely use PCE are more likely to state that PCE is harmless (Sweeney, 2010). The fact that users tend to estimate PCE usage as harmless might explain why they find its application to be morally and socially acceptable (Desantis and Hane, 2010).

Hence, there is a general discrepancy between the views of users and nonusers with regard to the associated health risks of PCE and its moral acceptance, with users being less concerned than nonusers.

## Coercion

The question of coercion relates to autonomy, i.e. the freedom to decide about one's personal life, and is a central issue in the normative debate on PCE. A main concern is that people are being pressured or even coerced into enhancing themselves. This might happen either indirectly in the form of peer pressure (Warren et al., 2009), or potentially directly in certain workplaces with long working hours and high demands on cognitive functioning, such as in the military or in surgery (Schoomaker, 2007; Maslen et al., in press). While opponents of enhancement consider it a “threat to the responsibility one bears for one's own life” (Habermas, 2003, p. 61), proponents instead focus on its advantages. They point out that PCE in particular entails the possibility of enhancing autonomy itself by increasing the reasoning abilities required to engage in such autonomous decisions (Schaefer et al., 2013).

The participants in Schuijff and Brom's (2013) focus group study indicated implicit peer pressure and explicit demand by employers to use human enhancement technologies to be a major concern for them. Maslen et al. (in press) found in a survey of 80 UK students clear and strong objection against the idea that people in professions with high responsibility, such as pilots and physicians, might even have a moral obligation to enhance their performance, with only one respondent agreeing to such an obligation and 44% completely disagreeing. Two other surveys among German students found that the majority of students did not approve of PCE in jobs with high responsibilities: in one survey only 26% of the 1026 respondents approved of the use of PCE in highly responsible jobs (Schildmann et al., 2013), the second found that approximate 20% of their 1547 respondents approved the use of PCE for individuals with high responsibility (Franke et al., 2012a).

Forlini and Racine (2009) conducted one of the (very few) studies investigating people's attitudes on autonomy and coercion specifically with regards to PCE, again with the use of focus groups. Their participants, 65 Canadians assigned to one of nine groups consisting of either students, parents, or healthcare providers, agreed that PCE should be a matter of personal choice. This seems in line with one of the recommendations of a “European citizens' panel” held in 2005 and 2006, a citizens' deliberation on brain science involving 126 individuals from nine European countries. (A citizen's deliberation is a form of public participation in consultation about science, but can be less structured than a focus group study because groups can change during the deliberation.) One of the topics touched upon briefly was human enhancement. The participants' highest ranked recommendation was that people should be given the right to take “whatever drug they want,” but enough information about the effects and dangers should be available; however, they did not support use of PCE in situations in which people have to pass exams (European Citizens Panel, 2006).

Participants in Forlini and Racine's (2009) study held the descriptive view that users are generally deciding to take such substances as a result of a voluntary decision. At the same time, however, they believed that this decision can be influenced by perceived social pressure or by competitive environments, such as academia or the job market, where people are striving for success and feel they have to perform better than average. Health care providers amongst the participants admitted that students who don't take enhancers may be disadvantaged because demands are getting higher and PCE is becoming more prevalent. They regarded peer pressure as an important contributing factor to the perceived need to take cognitive enhancers. Parents, in contrast, were aware of the pressure being put on students and consequently felt worry and sadness. They feared that the use of PCE may become a new standard.

However, peer pressure seems to be a more complex phenomenon than one might assume. Sattler et al. (2014) show that willingness to take PCE drugs does not increase when others encourage it, but it decreases when disapproval of the use of PCE by others rises. Furthermore, on the one hand, Forlini and Racine (2009) observe the desire of students not to be at a disadvantage, while on the other hand, less than 10% of 1547 German students stated in a survey by Franke et al. (2012a) that they would use PCE if others did so. In an online study amongst 6275 Swiss students less than 3% agreed that other people's use of substances would justify the use of PCE, compared to over 66% who agreed that increased learning would justify the use (Maier et al., 2013). One methodological reason for these diverging findings might be that even though a qualitative approach as used by Forlini and Racine (2009) can reveal aspects that might stay undiscovered in quantitative approaches like surveys, the small sample size might limit the generalizability of the findings. Going beyond methodology, we might speculate that it is not other people using PCE *per se*, but other people performing better, that puts pressure on students and leads them to consider taking such substances. In general, student participants in Forlini and Racine's (2009) focus groups viewed PCE as a personal lifestyle choice and emphasized the importance of personal integrity, i.e., they accepted the use of PCE conditional on the fact that one remains faithful to one's personal values. At the same time, they recognized the difficulty of that endeavor when social pressure is high and when abstinence could lead to personal disadvantage. Parents of university students, on the other hand, maintained a paternalistic view: students should be informed about cognitive enhancement, and, as a consequence, they should be held responsible and accountable for the decision to engage in PCE.

Thus, people consider the role of peer pressure as problematic and agree on the importance of deciding autonomously whether to engage in PCE. However, since the few studies reported here reveal mixed results, more research is needed to investigate the topic in greater depth.

## Fairness

The normative debate around the fairness of PCE is perhaps the least clearly defined. The term “fairness” seems to raise different distinct concepts in the lay mind, and thus creates a difficulty in comparing different studies that ask for opinions on the fairness of PCE use without defining what is meant by “fair.” Overall, fairness related concerns seem to play an important role in the public, since they have been the second most common argument, after unintended side effects, against the use of enhancement raised by participants in Santoni de Sio et al.'s (in press) survey. Forlini and Racine's (2012a also c.f. Forlini and Racine, 2009) focus-group study explored lay statements about fairness of PCE in greater detail. They developed a model to describe three different subthemes: they suggest that judgments of fairness can (apart from external factors like legislation) be defined by a relationship between *equality of opportunity*, *honesty*, and *authenticity*. Participants who valued the equality of opportunity described the importance of an equal distribution of opportunities to obtain PCE substances and opportunities deriving from their use. Honesty and authenticity are both related to effort that has to be invested to achieve a certain task. The underlying assumption is that when high performance is achieved with less effort—as might be the case when PCE substances are taken—this might be less fair compared to performance that is achieved with substantial effort. Honesty relates to the social aspect of this assumption and reflects the effect of PCE use on other individuals, for example in a competitive environment where PCE use might be seen as cheating. Authenticity relates to the individual PCE user and questions whether his/her performance under PCE, often seen as a situation in which effort is discounted, is an authentic performance. It is based on the underlying belief that putting in effort shapes the experience of an individual and thus affects a “future” individual that does not gain the same experience while using PCE. Although the above separation of concepts is too coarse to fully reflect the depth of the academic normative debate, the concerns of the public can be grouped around these subthemes of fairness.

### Equality of opportunity

First, we discuss equality of opportunity, the fear that inequality in access to enhancement substances might increase inequalities in society. Farah et al. (2004) describe how certain groups might experience cost barriers and social barriers to access PCE. (In this section, we do not consider concerns about restrictions of freedom to follow personal preferences, as these are discussed in the previous section about coercion). Equality of opportunity is also referred to as *distributive fairness* (e.g., Scheske and Schnall, 2012), *distributive justice* (Farah et al., 2004), or the *concern of inequality* (e.g., Bostrom and Sandberg, 2009). Note that the notion of equality of opportunity relates to a certain underlying theory of justice (cf. Rawls, 1971), which can be contrasted, for example, to the notion of equality of outcome. However, these underlying theories are not distinguished yet in empirical research on the public's opinions about PCE.

Although healthcare providers, students and parents who participated in the focus group study by Forlini and Racine (2012a) believed that, currently, everybody who wanted could find PCE substances one way or another, they did emphasize the importance of the value of equality of opportunity as part of their judgment on the fairness of the use of PCE. Correspondingly, in survey research by Sattler et al. (2013a) amongst 1852 respondents, a lower score on both willingness-to-use a PCE and moral acceptability of PCE substances was reported when judging an imaginary situation where no other students take this PCE substance, compared to situations in which half of the other students or all fellow students were taking the PCE substance. Possibly, they read the “no other student” situation as one where there is inequality in opportunity with them having, and other students not having, access and found this to be morally unacceptable. However, Sattler et al.'s (2013a) finding could also point to the experience of a “social norm,” following from the prevalence of use that influences the judgment of moral acceptability.

In a survey, Hotze et al. (2011) presented 633 US American general practitioners with two statements related to this topic: a slight majority agreed that society should prevent economic advantages turning into biological advantages (57%), and that everyone should have equal access to medical enhancers (55%). Scheske and Schnall (2012) showed that the use of PCE substances is perceived as more wrong if not everybody can afford them, compared to situations in which everybody can. Fitz et al. (2013) investigated fairness by using the contrastive vignette technique online. Their 4011 respondents, recruited via Amazon's Mechanical Turk, were randomly assigned to one of 22 different vignettes that described the use of PCE diverging in terms of alleged safety, societal and peer pressure, fairness and authenticity. Respondents saw it as less fair if a student obtained an enhancer with the help of money given by his parents rather than with money earned by own work. The 185 US American respondents in Sabini and Monterosso's (2005) study endorsed the so-called “interaction view” (p. 91): their judgments of fairness depended on the group that was affected by the drug. Although their fairness ratings of PCE use were always close to or lower than the midpoint of the scale—thus generally regarding it as rather unfair—respondents believed the use by the worst performing 10% of students to be fairer than the use by everybody or the top 10%. The “interaction view” corresponds to what John Rawls (1971, 1985) calls the “difference principle”: inequality is acceptable only if the current situation for those least advantaged is improved.

A survey amongst 1026 German students demonstrated that 27% of the respondents approved the use of PCE by worse performing classmates, while 57% approved the use for elderly with declining cognitive performance (Schildmann et al., 2013). Correspondingly, over a quarter of the respondents of another study among German students reported that classmates with low academic performance should be allowed to use PCE, while 50% indicated that the use of PCE by cognitively impaired elderly should be allowed (Franke et al., 2012a). The percentage of respondents who endorsed the use of PCE by classmates with low academic performance was higher among PCE users than nonusers. It is clear that although the interaction view is endorsed, previous performance is of less influence on judgments of fairness of the use of PCE than age. However, this is perhaps because the samples are both young students who are in competition with other young students, so that age and performance variables could be said to be confounded in the sample. Future studies need to investigate the attitudes of young students in competition with elderly students, and compare elderly students with elderly non-students, so that attitudes toward these factors could be separated. Sabini and Monterosso (2005) explain the interaction view by arguing that a substance that would affect the worst performing 10% only can better be seen as a normalizer instead of an enhancer, suggesting that in this case it might be closer to a treatment than to an enhancement. If this explanation is correct, it would seem that both the acceptance of side effects as well as the acceptance of a certain unequal distribution is greater in the case of a treatment compared to enhancement.

In general, healthcare providers, students, and parents seem to agree that an unequal distribution of PCE is unfair, if the unequal distribution is related to factors that are changeable, such as wealth. If the unequal distribution exists due to biological dispositions, such as having a low attention span, an unequal distribution is seen as less relevant to moral judgments. This is related to the distinction between treatment and enhancement, in which the former is generally believed to be seen as more acceptable (Parens, 1998; Daniels, 2000; Hyman, 2011). Users find it more acceptable than nonusers that fellow students with low academic performance use PCE, but research investigating the reasons for these diverging views of users and nonusers is still lacking.

### Honesty

Honesty relates to the question of whether the use of PCE might give a user an unfair advantage over people who do not use PCE, and thus might need to exert more effort to achieve the same result. Scheske and Schnall (2012) refer to honesty as *competitive fairness* and Bostrom and Sandberg (2009) discuss it in relation to *cheating*. Using an example and taking a normative stance, Bostrom and Sandberg (2009) describe how goals and rules define whether an act qualifies as cheating: if the primary goal of schooling is acquiring knowledge, PCE might be legitimate. In the case of a competition for grades or admission, however, PCE could be seen as cheating if it were against the rules or if access were unequally distributed.

Quantitative accounts of the public's opinion on honesty related to PCE use can be found in several studies. Students of a highly competitive UK university deemed the competitive advantage PCE can give as one of the most important concerns regarding its use: an advantage due to PCE use was found most morally wrong when no other competitors were taking the substance, relating competitive to distributive fairness and peer pressure (Scheske and Schnall, 2012). Moreover, an online survey by Bossaer et al. (2013) demonstrated that 60% of the 372 student respondents agreed that PCE provides users with an unfair advantage over other students. An almost identical amount of just over half of the respondents (56%) believed that PCE use for study purposes could be seen as academic dishonesty. In a large-scale online survey, with respectively 5882 and 3486 participants in the first and second wave, (Dubljević et al., 2013) found that German students deem the use of PCE with the intention to increase study performance to be morally less acceptable than traditional forms of academic misconduct, such as cheating in exams, fabrication, or plagiarism. Schildmann et al. (2013) reported that half of the respondents of their questionnaire study thought the use of PCE by others was unfair, another quarter was unsure of their thoughts about the statement, and only a quarter “rather” or “absolutely” agreed that the use of PCE by others was fair. Over half of the respondents in Franke et al. (2012a) indicated in a questionnaire that they found PCE fair “under no circumstances” or “probably not.” This percentage was higher among females and among nonusers. Although exact percentages are not provided, Sweeney (2010) also discusses a survey amongst 100 students in which nonusers felt more troubled than users by academic advantages obtained by PCE. This difference between users and nonusers was also found in the online survey by Ott and Biller-Andorno (2013). They show that a little over 40% of nonusers agree that with using PCE, one would be betraying others who do not use PCE, while less than 20% of users agree to this statement. An online study with 1200 male US American student participants revealed that the misuse of performance enhancement substances in the sporting domain received a higher rating on a scale that measured the degree of cheating than the use of PCE in academia (Dodge et al., 2012).

As described above, Fitz et al. (2013) showed that when obtaining PCE takes less effort, it is seen as more unfair. This was reflected in reduced fairness ratings in a scenario in which one individual could use PCE and another could not. The effort needed to obtain PCE was manipulated, as well as a second variable, that of the effect of the PCE as either reducing the effort needed to study or increasing the number of hours that one could study for. The combination of diminished effort in obtainment and reduced study effort produced the lowest ratings of fairness. Thus, any variation of the description that would result in a reduction of effort for the user of PCE in comparison to a nonuser resulted in respondents judging PCE as less fair.

Qualitative studies provide a more elaborate but also more ambiguous perspective on honesty in relation to PCE. In an interview study amongst 19 Australian students by Bell et al. (2013 also c.f. Partridge et al., 2013) fairness appeared at the top of the list of concerns mentioned. More than half of the participants described PCE as a form of cheating, while most of the others explicitly reported that they did not find it unfair in comparison to other available methods for performance improvement (e.g., coffee). In contrast to coffee, focus group participants saw PCE as a form of cheating similar to the use of steroids (Forlini and Racine, 2012b). Also, participants showed dissent and indecision about whether PCE use should be seen as cheating or not (Forlini and Racine, 2012a). Each subgroup of students, parents, and health care providers included some individuals who saw PCE as an unfair shortcut, putting others at a disadvantage, but also individuals who considered PCE a study tool like any other.

To summarize, a little over half of the public believes that the use of PCE provides an unfair advantage to users, a situation that is seen as cheating, especially in highly competitive environments. Nonusers provide lower ratings of honesty than users in quantitative studies. In general there seems to be dissent on whether PCE qualifies as cheating or not. Qualitative studies give more insight into the different perspectives on this question.

### Authenticity

In the normative debate, critics of PCE argue that users, compared with nonusers, of enhancement substances experience the value of exerting effort on a task less. This makes activities less meaningful and facilitates fewer experiences of self-development (see Schermer, 2008, for an analysis of this argument). Part of the debate therefore relates to authentic performance, for which effort is seen as a necessary condition (e.g., President's Council on Bioethics, 2003). Goodman (2010) specifies that this is true for what he calls process goods, for which the activity itself is seen as central. For so-called outcome goods, however, for which the result of an activity counts, effort is less relevant.

We can apply the distinction between process goods and outcome goods to the list of 19 traits rated by 357 respondents in an online study by Riis et al. (2008). For each trait they had to rate their willingness to enhance it using pharmacological means, if they could. Outcome related traits, such as memory, received higher scores on willingness-to-enhance than more process related traits, such as mood and social comfort. These higher ratings for what we deem outcome related traits might be explained by respondents valuing the notion of effort. Furthermore, respondents were less willing to enhance traits that were rated as more fundamental to the self, such as kindness and empathy.

Participants in the focus group study by Forlini and Racine (2012a) explicitly related effort to judgments of the fairness of PCE. Students, parents, and healthcare providers commented on effort and authenticity. They displayed both positive (non-problematizing) and negative (problematizing) views toward PCE in relation to effort and authenticity in equal proportions, as can be seen in the following examples. Participants of each target group (students, parents, and healthcare providers) noted that even with the use of PCE, effort still had to be put in to complete certain tasks. However, participants also discussed how enhancement may compromise certain social and personal values that shape an individual's behavior. Also some of the student participants in the interview study by Bell et al. (2013) described PCE as a quick fix rather than a true reflection of an individual's abilities, while other participants neither reported effort nor authenticity as being corrupted by the use of PCE. Two thirds of the student respondents in the survey by Schildmann et al. (2013) agreed that performances done with PCE were “less commendable than comparable performances that are done without these substances” (p. 25). In addition, 63% of the 644 physicians who completed the survey by Hotze et al. (2011) viewed PCE as a threat to the value of human achievement. Moreover, an online survey by Banjo et al. (2010) showed that the respondents, 212 physicians, ranked “PCE undermines the values of personal effort” as the fourth most important reason (of 13 presented) to feel uncomfortable about prescribing PCE to non-elderly people. However, an online survey among 1729 Australian students showed that users of prescription amphetamines had fewer concerns regarding the integrity of authentic and moral actions than did nonusers (Mazanov et al., 2013).

Finally, in their experimental vignette study Fitz et al. (2013) found that respondents rated an individual's performance as significantly less authentic when PCE was used, compared to when it was not used. However, this opinion did not completely transfer to judgments of worthiness for a promotion when the character would be assigned a new project in his job. Respondents judged a PCE user as significantly more worthy when successful without enhancement compared to with enhancement, but they also indicated that people who succeeded with the help of PCE were more worthy of promotion than people who did not use PCE and failed.

To summarize, in general people are more likely to enhance outcome related traits than process related traits. However, in direct discussions about effort and authenticity in relation to fairness, individuals display divergent opinions on the importance of these topics. The proportion of people who believe effort is discounted and authenticity violated when using PCE is a little over half of the respondents in most studies. This implies that little less than half of the public is not that concerned about effort and authenticity in relation to PCE. One reason that is given is that effort still has to be invested to achieve certain goals, even when PCE is used.

## Conclusion

This review provides an overview of 40 studies on the public's opinion about PCE. Our main finding is that in groups outside the normative academic debate several concerns about the use of pharmacological substances for performance enhancement are either raised or endorsed. These concerns that are discussed in the current review of research on opinions about PCE widely match the normative debate. These similarities between the public's opinion and the normative debate are also found in studies on other ethical issues, for example the sex selection of embryos (Banks et al., 2006) and life extension technologies (Partridge et al., 2009). The findings we present are divided between three concerns regarding PCE use which are common in both the ethical and lay debate: medical safety, coercion, and fairness.

Several studies have shown that *medical safety* is regarded as central by nonusers of PCE, and insecurity about it provides a reason for them to refuse the use of PCE. Related to this concern are findings that point toward a preference for natural over artificial enhancers. A similar preference can be seen for interventions that might be closer to treatment than to enhancement. Users, on the other hand, do not display these preferences and indicate conflicting results on judgments of (subjective) health risks associated with PCE. They are concerned about addiction, but do not worry about other health risks, and deem PCE more often harmless than nonusers do. A more convergent view can be found on the theme of *coercion*. Different subgroups agree that PCE should be a matter of personal choice. They believe that decisions concerning use are, in general, made voluntarily, although they can be influenced by perceived social pressure or by competitive environments. It is shown that peer pressure is a complex phenomenon, as students might not always be influenced by other people's PCE use itself, but only when these others achieve a higher performance compared to their own. However, only a few studies have investigated coercion to date and we call for future research to fill this gap. Finally, we discussed *fairness*, divided into three subthemes: equality of opportunity, honesty, and authenticity. An unequal distribution of PCE substances that might develop due to changeable factors—such as wealth—is seen as unfair, while an unequal distribution due to biological dispositions—such as a low attention span—is seen as less relevant to judgments of fairness. This might relate to a general finding that treatments are seen as more acceptable than enhancements. The public's opinion on the subthemes of honesty and authenticity shows a more complex pattern. Nonusers believe more often than users that the use of PCE provides an unfair advantage, although in general only half of the public raises concerns about this topic of honesty and cheating. Several studies show that around the theme of authenticity both problematizing and non-problematizing views on PCE arise in equal proportions. While some respondents indicate that “the work has still to be done” even when PCE is used, others believe that PCE is a quick fix and undermines an authentic performance.

One important distinction within the public can be found between users and nonusers, who tend to differ in their perspectives on the medical safety and subthemes of the fairness of PCE: while users generally deem PCE to be safe and fair, nonusers do not. These results imply that users are either more willing to engage in PCE because of their positive attitude toward it, or that they adopted their positive attitude as a result of personal usage. In either case, the differences in users' and nonusers' attitudes toward PCE might be driven by cognitive biases. It is possible, for example, that nonusers display a more negative view toward PCE because they experience the so-called status quo bias, which describes the irrational preference for an option only because it preserves the current state of affairs (Kahneman and Tversky, 2000). As currently PCE use is not seen as a common way to improve cognitive performance, this bias might result in the preference to not use PCE. This tendency, to use the status quo as a reference point, might explain why people prefer interventions when they are seen as treatments as opposed to enhancements (Bostrom and Ord, 2006). It is also possible, however, that users adopt a more positive view toward PCE in order to reduce their cognitive dissonance, the discomfort experienced when one's actions don't reflect one's beliefs (Festinger, 1957). This would reflect a situation in which users adapt their attitudes toward their PCE use, that is, their behavior. Future research is called to examine in greater depth which biases might influence people's attitudes toward PCE and the causal direction explaining the attitudes currently prevalent. Further, future research might also reveal whether differences between users and nonusers also hold for other concerns, such as coercion and authenticity for which data on this distinction is currently lacking.

It is important to note that the current literature focuses mostly on opinions of students, with only a few exceptions provided by studies on health care providers and the general public. Furthermore, several studies were conducted with a non-random sample of participants, or obtained a low response rate in the case of random sampling. This might have biased the results. In addition, even the studies that were conducted with random sampling of students at specific universities can create a biased population overview in our review, because differences between colleges on the use of PCE are found (e.g., McCabe et al., 2005). Furthermore, future research should provide more insight into the opinions of populations other than students, such as the general population or more specifically people active in the workforce. This would add to a more accurate picture of opinions of the general population and of potential users in those areas where use is to be expected.

Moreover, future research is called for to reveal more fine-grained differences in the public opinion for certain concerns. In the current literature, different concerns can have the same name, as is seen with fairness, while different names may also be used for the same concern, as was described for equality of opportunity. This makes it harder to draw precise conclusions on the state of research on public attitudes toward PCE and to systematically compare it with arguments from the normative debate. Although more fine-grained studies are needed to reflect the depth of the normative debate, it can be said that, thus far, concerns of the public regarding the use of PCE reflect the main issues fiercely debated in academia.

### Conflict of interest statement

The authors declare that the research was conducted in the absence of any commercial or financial relationships that could be construed as a potential conflict of interest.
